# Survey of Citizens’ Preferences for Combined Contact Tracing App Features During a Pandemic: Conjoint Analysis

**DOI:** 10.2196/53340

**Published:** 2024-11-14

**Authors:** Seiji Bito, Yachie Hayashi, Takanori Fujita, Ikuo Takahashi, Hiromi Arai, Shigeto Yonemura

**Affiliations:** 1 National Hospital Organization Tokyo Medical Center Tokyo Japan; 2 Department of Health Policy and Management School of Medicine Keio University Tokyo Japan; 3 Komazawa Legal Chambers Tokyo Japan; 4 RIKEN Center for Advanced Intelligence Project Toyko Japan; 5 The Graduate Schools for Law and Politics University of Tokyo Toyko Japan

**Keywords:** digital contact tracing apps, infectious disease, conjoint analysis, user attitudes, public preferences, citizen values, attitude to health, COVID-19, contact tracing, privacy, questionnaires

## Abstract

**Background:**

During the COVID-19 pandemic, an increased need for novel solutions such as digital contact tracing apps to mitigate virus spread became apparent. These apps have the potential to enhance public health initiatives through timely contact tracing and infection rate reduction. However, public and academic scrutiny has emerged around the adoption and use of these apps due to privacy concerns.

**Objective:**

This study aims to investigate public attitudes and preferences for contact tracing apps, specifically in Japan, using conjoint analysis to examine what specifications the public values most in such apps. By offering a nuanced understanding of the values that citizens prioritize, this study can help balance public health benefits and data privacy standards when designing contact tracing apps and serve as reference data for discussions on legal development and social consensus formation in the future.

**Methods:**

A cross-sectional, web-based questionnaire survey was conducted to determine how various factors related to the development and integration of infectious disease apps affect the public’s intention to use such apps. Individuals were recruited anonymously by a survey company. All respondents were asked to indicate their preferences for a combination of basic attributes and infectious disease app features for conjoint analysis. The respondents were randomly divided into 2 groups: one responded to a scenario where the government was assumed to be the entity dealing with infectious disease apps (ie, the government cluster), and the other responded to a scenario where a commercial company was assumed to be this entity (ie, the business cluster). Samples of 500 respondents from each randomly selected group were used as target data.

**Results:**

For the government cluster, the most important attribute in scenario A was distributor rights (42.557), followed by public benefits (29.458), personal health benefits (22.725), and profit sharing (5.260). For the business cluster, the most important attribute was distributor rights (45.870), followed by public benefits (32.896), personal health benefits (13.994), and profit sharing (7.240). Hence, personal health benefits tend to be more important in encouraging active app use than personal financial benefits. However, the factor that increased motivation for app use the most was the public health benefits of cutting infections by half. Further, concern about the use of personal data collected by the app for any secondary purpose was a negative incentive, which was more significant toward app use compared to the other 3 factors.

**Conclusions:**

The findings suggest that potential app users are positively motivated not only by personal health benefits but also by contributing to public health. Thus, a combined approach can be taken to increase app use.

## Introduction

The COVID-19 pandemic introduced significant challenges to societies globally, necessitating novel solutions such as digital contact tracing apps to mitigate the spread of the SARS-CoV-2 virus [1-3]. Digital contact tracing apps may potentially enhance public health initiatives through timely contact tracing and infection rate reduction. However, privacy concerns have led to public and academic scrutiny over the adoption and use of these apps [4,5].

In the context of digital contact tracing, several studies have examined dilemmas related to privacy, trust, and the potential for coercion [4,6-8]. A thorough review of 21 contact tracing apps highlighted the difficulty in balancing data privacy standards with public health benefits [9]. Furthermore, a Europe-wide survey revealed public anxiety about privacy issues associated with these apps [4,10], aligning with broader health policy debates, particularly those surrounding ethical implications. Trust, transparency, and clear communication have been emphasized as essential to ensuring public confidence in these technologies [11].

COVID-19 Contact-Confirming Application (COCOA) is a smartphone app released by Japan’s Ministry of Health, Labour and Welfare (MHLW) that underscores respect for privacy and minimal handling of personal information, contrasting with the infectious disease countermeasures with privacy risks undertaken by countries such as Singapore and South Korea [12,13]. However, criticism has arisen that COCOA has had a limited effect in helping to prevent the spread of infectious diseases [14]. The friction between infectious disease countermeasures, ongoing economic activities, and respect for privacy remains a pertinent issue.

We examined a survey questioning people’s public attitudes toward apps such as COCOA in 2022 [15]. The study indicated that people may place greater emphasis on social significance, such as public health improvement, rather than economic incentives. However, the order of preference regarding specific app requirement specifications remains largely unclear.

This study aims to build on existing knowledge by investigating public attitudes and preferences for contact tracing apps in Japan by using “conjoint analysis” to examine what specifications the public values most in contact tracing apps. By offering a nuanced understanding of the values that citizens prioritize, this research can help balance public health benefits and data privacy standards in the design of contact tracing apps and potentially serve as reference data for discussions on legal development and social consensus formation in the future.

## Methods

### Participants

A cross-sectional, web-based questionnaire survey was conducted to determine how various factors related to the development and distribution of infectious disease apps affect the public’s intention to use such apps. The survey was open to the public, with no representative sample of the target population, and was conducted on the web using the survey monitors of the market research company, Cross Marketing Inc (CMI) [16]. We defined the target population for the survey as general consumers aged 18 years and over living in Japan who have their own mobile communication devices. We then commissioned CMI to use the following procedure for recruiting the target population for the survey: (1) CMI will conduct all aspects of the survey, from recruiting the target population and sampling to collecting the data on the web; (2) CMI will create a website dedicated to the survey, post the survey purpose statement formulated by the researcher on the website, and then advertise to the candidate survey respondents managed by CMI (the survey statement clearly stated that no identifying information would be collected); (3) from among the people who accessed the website, those who agreed to the purpose and method of the survey were asked to click on a link to participate in the survey; and (4) when the number of survey participants who agreed to participate exceeded 1000, they were randomly assigned to 2 response groups using a random number table.

One response group responded to a scenario in which the government was assumed to be the main entity dealing with infectious disease (hereafter referred to as the “government cluster”), whereas the other group responded to a scenario in which a commercial company was the main entity dealing with infectious disease (hereafter referred to as the “business cluster”). We divided the respondents into 2 groups under the assumption that the entity (ie, government or commercial company) that handles infectious disease apps would have an impact on the public’s acceptance of and attitudes toward infectious disease apps.

### Data Collection

To conduct a cross-sectional survey based on conjoint analysis, the respondents were directed to a survey website created by CMI, and their responses were collected. They were asked to answer questions about their basic characteristics, installation status of the contact verification app (COCOA), vaccination status, main sources of infection information, privacy concerns regarding national infection control measures, and history of novel coronavirus infections from a list of options. They were asked to rank their preferences for combinations of infectious disease apps in the order of their willingness to use each of the 9 combinations. Of the respondents who answered all questions and clicked the “Submit Response” button, a sample of 500 from each randomly selected group were received anonymously by CMI and used as target data. This survey was conducted in October 2022.

### Measurements

All respondents were asked to indicate their preferences for a combination of basic attributes and infectious disease app features for conjoint analysis. The basic attributes included biological sex, age group (in 10-year increments), occupation, most recent education, marital status, presence of a roommate, smartphone ownership, installation of COCOA, vaccination status, main source of infection information, privacy concerns regarding national infectious disease control measures, and previous experience with a novel coronavirus infection. Respondents were asked about their history of novel coronavirus infections. In addition, scenarios A and B were provided for the functionality of the infectious disease app, and respondents were asked to indicate which combination of features they would prefer to use in each of these scenarios Multimedia Appendix 1. In scenario A, when the app developer (either the MHLW or an internet retailer) invites the public to use a new free infectious disease app, they are also asked whether they would be willing to use the specific features of the existing infectious disease app, such as the function of notifying the public when coming into contact with a person who tested positive. In scenario B, when the organization invites the public to use a new free infectious disease app, the following functions are considered: promotion of the use of the infectious disease app by the public, notifications of infection risk status, notifications regarding the need for testing and the promotion of specific measures, information about priority appointments for testing and medical examinations, collection of personal information for public health measures, and secondary use of collected information. The specific functions were presented in addition to the function of the existing infectious disease app, which is the function of notifying the public when coming into contact with a person who tested positive. In scenario B, the registration of the respondent’s name, smartphone number, and email address would be required as a condition for registering to use the infectious disease app.

### Conjoint Design

Profit sharing was the first attribute in both scenarios A and B. Both scenarios investigated 4 attributes, each with 3 levels. Nine combinations generated using the *conjoint* procedure in SPSS (version 25; IBM Corp) were presented, and participants were asked to rearrange the attributes in their preferred order. They could do so as many times as they desired, using the drag-and-drop function on the website, until they had arrived at the order that they personally felt was optimal.

The 4 features used as attributes in scenario A were as follows: profit sharing (A-1), public benefits (A-2), personal health benefits (A-3), and distributor rights (A-4). Each feature had 3 levels. A-1 comprised 3 levels of profit sharing for the app user: no financial profit sharing (none), a JP ¥500 (a currency exchange rate of JP ¥140=US $1 is applicable) discount coupon per month (JP ¥500), and a JP ¥1500 discount coupon per month (JP ¥1500). A-2 comprised 3 levels of contribution to public health resulting from registering with the contact tracing app: unknown whether infection control measures will benefit (unknown), expected to cut infections by half (infections halved), and expected to cut infections and hospitalizations by half (infections + hospitalizations halved). A-3 comprised 3 levels of notification to the app user regarding health information: immediate notification to the app user if there was close contact with an infected person (contact notification), a priority appointment for a free polymerase chain reaction test in addition to contact notification (test appointment), and a priority appointment for a free test and examination if they tested positive (test + examination appointment). A-4 comprised 3 levels of authorization for the MHLW to use personal data outside of the app: not authorized to handle personal app user data (no authorization), authorized to use for data analysis in the context of infection control measures only and for notifications to the app user (infection control only), and authorized to use app user data for broad purposes outside of infection control measures, including advertising (broad use). The combinations of the attributes and levels used in scenario A are shown in Table 1.

The 4 features used as attributes in scenario B were: profit sharing (B-1), scope of personal data (B-2), scope of use (B-2), and third-party data sharing (B-2). Each feature had 3 levels. B-1 was identical to A-1. B-2 comprised 3 levels of the scope of the personal data that the MHLW would collect: data from registration only (registration data), registration data + data concerning location and time of contact with an infected person (contact location data), and registration data + a record of all movements (all movement data). B-3 comprised 3 levels of the scope of use for the personal data that were collected: for planning and implementing measures to prevent the spread of infection (infection control only), for addressing and disseminating information during a state of emergency due to earthquakes or other disasters in addition to the previous (infection + other public safety measures), and for notifications from businesses to app users about useful information and discounts, in addition, to the previous (public safety measures + advertising). B-4 comprised 3 levels of sharing of app user data with commercial businesses and other third parties by the app distributor: cannot provide app user data to third parties (data sharing prohibited), can provide data if anonymized so that individuals cannot be identified (sharing of anonymous data), and can provide data that identify individuals (sharing of personal data). The combinations of the attributes and levels used in scenario B are shown in Table 2.

**Table 1:** Attributes and levels of the app features in scenario A (JP ¥140=US $1).

Attributes	Levels
Profit sharing (profit gained by registering with the contact tracing app)	None JP ¥500 JP ¥1500
Public benefits (contribution to infection control measures through registering with the contact tracing app)	Unknown Infections halved Infections + hospitalizations halved
Personal health benefits (health information and opportunities for examination gained by individuals)	Contact notification Test appointment Test + examination appointment
Distributor rights (authorization to use app user data gained by the distributor of the contact tracing app)	No authorization Infection control only Broad use

**Table 2:** Attributes and levels of the app features in scenario B (JP ¥140=US $1).

Attributes	Levels
Profit sharing (profit gained by registering with the contact tracing app)	None JP ¥500 JP ¥1500
Scope of personal data (scope of the personal data collected by the distributor of the contact tracing app)	Registration data Contact location data All movement data
Scope of use (scope of use of the collected personal data)	Infection control only Infection + other public safety measures Public safety measures + advertising
Third-party data sharing (scope of the data that the distributor of the contact tracing app can share)	Data sharing prohibited Sharing of anonymous data Sharing of personal data

### Statistical Analysis

The distribution of respondent characteristics and attitudes toward COVID-19 infection prevention measures were described for the government and business clusters, respectively. For the conjoint analysis, the importance of each attribute was calculated for scenarios A and B individually. In addition, partial utility values were calculated for all levels of each attribute. For the partial utility values, the default level for each attribute was set to 0 and the utility value of each step level was calculated relative to that level.

The *conjoint* procedure in SPSS (version 25) was used to create the orthogonal table for the conjoint analysis of the combination of 4 attributes × 3 levels. For the level setting in the calculation of utility values, linear type variables were used for the profit return attribute of A-1 and B-2, while category type variables were used for all other attribute levels.

### Ethical Considerations

This study was reviewed and approved by the Biomedical Research Ethics Committee of the National Hospital Organization Tokyo Medical Center on September 5, 2022 (approval R22-051). This research and the research plan were designed in accordance with the Helsinki Declaration, the Belmont Report, and the “Ethical Guidelines for Human Subjects of Life Science and Medical Research” joint statement of the Japanese MHLW; the Ministry of Education, Culture, Sports, Science and Technology; and the Ministry of Economy, Trade and Industry. In the survey, the survey company CMI first provided the survey monitors with a research purpose statement prepared by the researchers, and the survey monitors gave their consent to participate in the survey after reading and understanding the statement. After the survey period ended, the response data sent by the survey participants were downloaded from the CMI’s website and stored on a storage medium that was disconnected from the internet. The data from the research company were received by the researchers as anonymous data with any identifying information removed. To prevent data leaks, the analysis was conducted only in the Clinical Epidemiology Laboratory at the National Hospital Organization Tokyo Medical Center. The researchers verified that the data set did not contain any information that could be used to identify individuals. We did not offer compensation to survey respondents for participating in the survey, as we considered any disadvantages that might arise from responding to the survey to be minimal.

## Results

### Demographics

Table 3 shows the respondents’ basic attributes and other descriptive statistics for the government and business clusters, respectively. The proportion of government and business clusters (n=500 each) that did not have COCOA installed were 64.8% (n=324) and 62.8% (n=314), respectively, while 12.4% (n=62) and 11.8% (n=59) had installed and were using COCOA. In the government and business clusters, 13.2% (n=66) and 12.4% (n=62) had not received any COVID-19 vaccinations, respectively, while roughly half of each cluster (n=237, 47.4% and n=248, 49.6%, respectively) had received 3 vaccinations. A total of 21% (n=105) of the government cluster and 21.2% (n=106) of the business cluster had or intended to receive a fourth vaccination or more.

Thereafter, participants were asked about the handling of infected persons’ personal data by the government, medical facilities, and private organizations for the purpose of preventing the spread of COVID-19 infections. The most common response regarding concerns over personal data leaks was “somewhat worried” (government cluster: n=229, 45.8%; business cluster: n=232, 46.4%), followed in descending order by “not really worried” (government cluster: n=135, 27%; business cluster: n=146, 29.2%), “very worried” (government cluster: n=94, 18.8%; business cluster: n=77, 15.4%), and “not worried” (government cluster: n=42, 8.4%; business cluster: n=45, 9%). Responses regarding concerns over government use for purposes other than those specified were, in descending order, “somewhat worried” (n=216, 43.2% for both government and business clusters), “not really worried” (government cluster: n=159, 31.8%; business cluster: n=158, 31.6%), “very worried” (government cluster: n=85, 17%; business cluster: n=76, 15.2%), and “not worried” (government cluster: n=40, 8%; business cluster: n=50, 10%). Responses regarding resistance toward the use of personal data by the government were, in descending order, “somewhat resistant” (government cluster: n=224, 44.8%; business cluster: n=208, 41.6%), “not really resistant” (government cluster: n=154, 30.8%; business cluster n=173, 34.6%), “very resistant” (government cluster: n=78, 15.6%; business cluster: n=66, 13.2%), and “not resistant” (government cluster: n=44, 8.8%; business cluster: n=53, 10.6%). However, when personal data use was by a hospital or other medical facility, including privately operated institutions, the responses were “somewhat resistant” (government cluster: n=204, 40.8%; business cluster: n=190, 38%), “not really resistant” (government cluster: n=188, 37.6%; business cluster: n=201, 40.2%), “very resistant” (government cluster: n=59, 11.8%, business cluster: n=50, 10%), and “not resistant” (government cluster: n=49, 9.8%; business cluster: n=59, 11.8%). Finally, when personal data use was by a private organization, including general businesses, the responses were “somewhat resistant” (government cluster: n=247, 49.4%; business cluster: n=231, 46.2%), “not really resistant” (government cluster: n=114, 22.8%; business cluster: n=140, 28%), “very resistant” (government cluster: n=98, 19.6%; business cluster: n=89, 17.8%), and “not resistant” (government cluster: n=41, 8.2%; business cluster: n=40, 8%). Thus, respondents felt less resistance toward medical facilities than toward the government, but more resistance toward private organizations than toward the government.

Concerning the experience of contracting COVID-19, 84.4% (n=422) and 85.6% (n=428) of the government and business clusters had never been infected, 11.4% (n=57) and 10.4% (n=52) had been infected and shown symptoms, 2.6% (n=13) and 3% (n=15) had been infected but asymptomatic, and 1.6% (n=8) and 1% (n=5) had been hospitalized due to infection, respectively.

**Table 3:** Respondents’ demographic distributions and attitudes toward the handling of one’s personal information by cluster for the purpose of preventing the spread of COVID-19 infections.

Characteristics		Government cluster (n=500), n (%)	Business cluster (n=500), n (%)
**Sex**			
	Female	258 (51.6)	237 (47.4)
	Male	242 (48.4)	262 (52.4)
	Other	0 (0)	1 (0.2)
**Age group (years)**			
	18-24	93 (18.6)	83 (16.6)
	25-34	102 (20.4)	109 (21.8)
	35-44	91 (18.2)	90 (18)
	45-54	76 (15.2)	71 (14.2)
	55-64	69 (13.8)	68 (13.6)
	65-74	42 (8.4)	53 (10.8)
	75 and older	27 (5.4)	26 (5.2)
**Occupation**			
	Company employee	202 (40.4)	210 (42)
	Government employee	19 (3.8)	25 (5)
	Independent business	20 (4)	14 (3)
	Company executive	7 (1.4)	3 (0.6)
	Self-employed	13 (2.6)	11 (2.2)
	Housewife or househusband	69 (13.8)	59 (11.8)
	Student	41 (8.2)	42 (8.4)
	Part-time work	43 (8.6)	46 (9.2)
	Side job	17 (3.4)	17 (3.4)
	Unemployed	69 (13.8)	73 (14.6)
**Educational attainment**			
	Elementary school	0 (0)	0 (0)
	Middle school	7 (1.4)	11 (2.2)
	High school or equivalent	126 (25.2)	120 (24)
	Junior college or technical school	77 (15.4)	66 (13.2)
	College	248 (49.6)	258 (51.6)
	Graduate school	30 (6)	30 (6)
	Other	12 (2.4)	15 (3)
**Marital status**			
	Married	240 (48)	239 (47.8)
	Unmarried	260 (52)	261 (52.2)
**Cohabitation status**			
	Living alone	108 (21.6)	122 (24.4)
	2-person household	137 (27.4)	130 (26)
	3-or-more person household	255 (51)	248 (49.6)
**COCOA^a^ installation**			
	Not installed	324 (64.8)	314 (62.8)
	Installed, but deleted	76 (15.2)	72 (14.4)
	Installed, but not using	38 (7.6)	55 (11)
	Installed and using	62 (12.4)	59 (11.8)
**COVID-19 vaccination status**			
	No vaccinations	66 (13.2)	62 (12.4)
	1 vaccination	2 (0.4)	3 (0.6)
	2 vaccinations	77 (15.4)	72 (14.4)
	3 vaccinations	237 (47.4)	248 (49.6)
	4 vaccinations (including planned vaccinations)	105 (21)	106 (21.2)
	Do not want to answer	13 (2.6)	9 (1.8)
**Fear of personal data leaks**			
	Not worried	42 (8.4)	45 (9)
	Not really worried	135 (27)	146 (29.2)
	Somewhat worried	229 (45.8)	232 (46.4)
	Very worried	94 (18.8)	77 (15.4)
**Fear of personal data use by the government** **for purposes other than those specified**			
	Not worried	40 (8)	50 (10)
	Not really worried	159 (31.8)	158 (31.6)
	Somewhat worried	216 (43.2)	216 (43.2)
	Very worried	85 (17)	76 (15.2)
**Resistance toward use of personal data by the government**			
	Not resistant	44 (8.8)	53 (10.6)
	Not really resistant	154 (30.8)	173 (34.6)
	Somewhat resistant	224 (44.8)	208 (41.6)
	Very resistant	78 (15.6)	66 (13.2)
**Resistance toward use of personal data** **by medical (including private) facilities for infection control**			
	Not resistant	49 (9.8)	59 (11.8)
	Not really resistant	188 (38)	201 (40.2)
	Somewhat resistant	204 (40.8)	190 (38)
	Very resistant	59 (11.8)	50 (10)
**Resistance toward use of personal data** **by private organizations for infection control**			
	Not resistant	41 (8.2)	40 (8)
	Not really resistant	114 (22.8)	140 (28)
	Somewhat resistant	247 (49.4)	231 (46.2)
	Very resistant	98 (19.6)	89 (17.8)
**History of contracting COVID-19**			
	Never infected	422 (84.4)	428 (85.6)
	Infected, no symptoms	13 (2.6)	15 (3)
	Infected, with symptoms	57 (11.4)	52 (10.4)
	Infected and hospitalized	8 (1.6)	5 (1)

^a^COCOA: COVID-19 Contact-Confirming Application.

### Importance Scores (Attributes)

For the government cluster, the most important attribute in scenario A was authorization for the MHLW to use the collected personal data for purposes outside of the app’s functions (distributor rights), followed by contributions to public health resulting from registering with the app (public benefits), notifications to the app user regarding health information (personal health benefits), and profit sharing for the app user (profit sharing). The importance scores were 42.557 for distributor rights, 29.458 for public benefits, 22.725 for personal health benefits, and 5.260 for profit sharing. In scenario B, participants were asked about their preferences when users were required to register their name, smartphone number, and email address during app registration. The most important attribute was the sharing of app user data with commercial businesses and other third parties by the MHLW (third-party data sharing), followed by profit sharing, the scope of the personal data collected by the MHLW (scope of personal data), and scope of use of the collected personal data (scope of use). The importance scores were 53.044 for third-party data sharing, 20.726 for profit sharing, 14.988 for scope of personal data, and 11.241 for scope of use (Tables 4 and 5).

For the business cluster, the most important attribute in scenario A was distributor rights, followed by public benefits, personal health benefits, and profit sharing. The importance scores were 45.870 for distributor rights, 32.896 for public benefits, 13.994 for personal health benefits, and 7.240 for profit sharing. In scenario B, as in the government cluster, participants were asked about their preferences when users were required to register their name, smartphone number, and email address when registering for the app. The most important attribute was third-party data sharing, followed by profit sharing, scope of personal data, and scope of use. The importance scores were 52.670 for third-party data sharing, 25.629 for profit sharing, 13.297 for scope of personal data, and 8.405 for scope of use (Tables 6 and 7).

**Table 4:** Importance scores by attribute in government cluster scenario A.

Attribute	Score or value	*P* value
Profit sharing	5.260	N/A^a^
Public benefits	29.458	N/A
Personal health benefits	22.725	N/A
Distributor rights	42.557	N/A
Pearson *r*	0.993	<.001
Kendall *τ*	0.944	<.001

^a^N/A: not available.

**Table 5:** Importance scores by attribute in government cluster scenario B.

Attribute	Score or value	*P* value
Profit sharing	20.726	N/A^a^
Scope of personal data	14.988	N/A
Scope of use	11.241	N/A
Third-party data sharing	53.044	N/A
Pearson *r*	0.944	<.001
Kendall *τ*	0.833	<.001

^a^N/A: not available.

**Table 6:** Importance scores by attribute in business cluster scenario A.

Attribute	Score or value	*P* value
Profit sharing	7.240	N/A^a^
Public benefits	32.896	N/A
Personal health benefits	13.994	N/A
Distributor rights	45.870	N/A
Pearson *r*	1.000	<.001
Kendall *τ*	1.000	<.001

^a^N/A: not available.

**Table 7:** Importance scores by attribute in business cluster scenario B.

Attribute	Score or value	*P* value
Profit sharing	25.629	N/A^a^
Scope of personal data	13.297	N/A
Scope of use	8.405	N/A
Third-party data sharing	52.670	N/A
Pearson *r*	0.977	<.001
Kendall *τ*	0.778	.002

^a^N/A: not available.

### Partworth Utilities (Levels)

**Figure 1:**
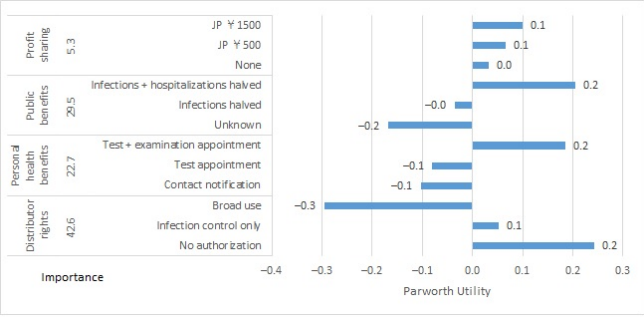
Importance and partworth utility values by levels of the attributes for government cluster scenario A. JP ¥140=US $1.

**Figure 2:**
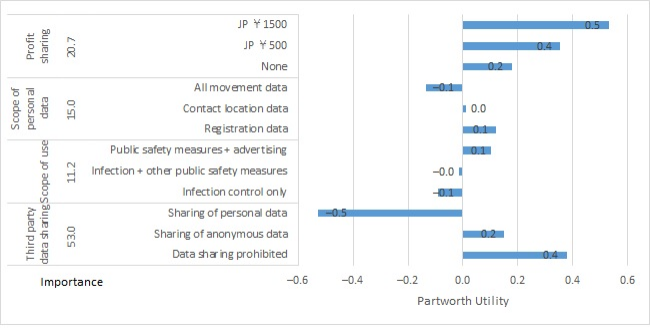
Importance and partworth utility values by levels of the attributes for government cluster scenario B. JP ¥140=US $1.

For the government cluster, partworth utilities (SE) for scenario A were as follows: for profit sharing, partworth utilities were +0.033 (0.045) for no financial profit sharing (none), +0.067 (0.090) for JP ¥500/month discount coupon (JP ¥500), and +0.100 (0.135) for JP ¥1500/month discount coupon (JP ¥1500). For public benefits, partworth utilities (SE) were –0.169 (0.052) for not knowing whether infection control measures would benefit (unknown), –0.036 (0.052) for expected to cut infections by half (infections halved), and +0.205 (0.052) for expected to cut infections and hospitalizations by half (infections + hospitalizations halved). For personal health benefits, partworth utilities (SE) were 0.103 (0.052) for immediate notification to the app user if there was close contact with an infected person (contact notification), –0.081 (0.052) for a priority appointment for a free polymerase chain reaction test in addition to contact notification (test appointment), and +0.185 (0.052) for a priority appointment for a free test and examination if testing positive in addition to the above (test + examination appointment). For distributor rights, partworth utilities (SE) were +0.243 (0.052) for not being authorized to handle personal app user data (no authorization), +0.053 (0.052) for being authorized for data analysis use cases in the context of infection control measures only and notifications to the app user (infection control only), and –0.296 (0.052) for being authorized to use app user data for broad purposes outside of infection control measures including advertisements (broad use; Figure 1). For scenario B, partworth utilities (SE) for profit sharing were +0.177 (0.184) for no financial profit sharing (none), +0.354 (0.368) for JP ¥500/month discount coupon (JP ¥500), and +0.531 (0.553) for JP ¥1500/month discount coupon (JP ¥1500). For scope of personal data, partworth utilities (SE) were +0.122 (0.213) for data from registration only (registration data), +0.012 (0.213) for registration data and data concerning location and time of contact with an infected person (contact location data), and –0.134 (0.213) for registration data and a record of all movements (all movement data). For scope of use, partworth utilities (SE) were –0.089 (0.213) for planning and implementing measures for preventing the spread of infection (infection control only), –0.015 (0.213) for addressing and disseminating information during a state of emergency due to earthquake or other disasters in addition to the previous (infection + other public safety measures), and +0.103 (0.213) for notifications from businesses to app users about useful information and discounts in addition to the previous (public safety measures + advertising). For third-party data sharing, partworth utilities (SE) were +0.378 (0.213) for not providing app user data to third parties (data sharing prohibited), +0.150 (0.213) for providing data if anonymized in order that individuals cannot be identified (sharing of anonymous data), and –0.528 (0.213) for providing data that identify individuals (sharing of personal data; Figure 2).

**Figure 3:**
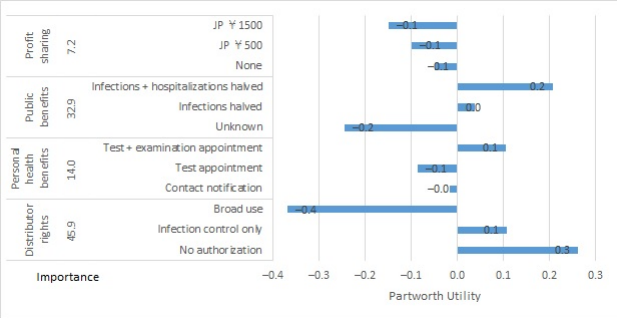
Importance and partworth utility values by levels of the attributes for business cluster scenario A. JP ¥140=US $1.

**Figure 4:**
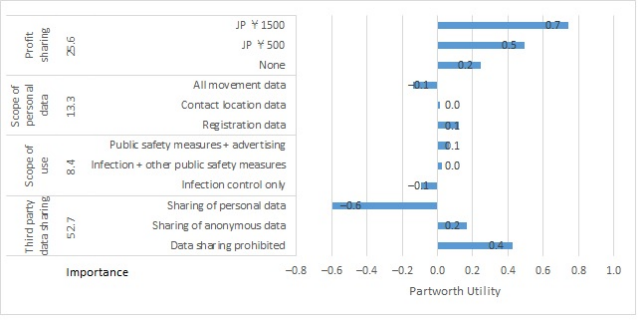
Importance and partworth utility values by levels of the attributes for business cluster scenario B. JP ¥140=US $1.

For the business cluster, partworth utilities (SE) for scenario A were as follows: for profit sharing, –0.050 (0.011) for none, –0.099 (0.022) for JP ¥500, and –0.149 (0.033) for JP ¥1500. For public benefits, partworth utilities (SE) were –0.244 (0.013) for unknown, +0.037 (0.013) for infections halved, and +0.207 (0.013) for infections + hospitalizations halved (Figure 3). For personal health benefits, partworth utilities (SE) were –0.017 (0.013) for contact notification, –0.087 (0.013) for test appointments, and +0.105 (0.013) for test + examination appointments. For distributor rights, partworth utilities (SE) were +0.261 (0.013) for no authorization, +0.107 (0.013) for infection control only, and –0.368 (0.013) for broad use. For scenario B, partworth utilities (SE) for profit sharing were +0.248 (0.133) for none, +0.496 (0.266) for JP ¥500, and +0.744 (0.398) for JP ¥1500. For scope of personal data, partworth utilities (SE) were +0.121 (0.153) for registration data, +0.016 (0.153) for contact location data, and –0.137 (0.153) for all movement data. For scope of use, partworth utilities (SE) were –0.095 (0.153) for infection control only, +0.028 (0.153) for infection + other public safety measures, and +0.067 (0.153) for public safety measures + advertising. For third-party data sharing, partworth utilities (SE) were +0.425 (0.153) for data sharing being prohibited, +0.169 (0.153) for sharing anonymous data, and –0.594 (0.153) for sharing personal data (Figure 4).

## Discussion

### Principal Findings

The previous study of contact tracing app preferences among the Japanese public examined the extent to which citizens felt resistance toward the use of the contact tracing app COCOA, which was used in Japan during the COVID-19 pandemic when various features were added. Specifically, these features were (1) automatic registration in an MHLW database for those who tested positive, (2) location data tracking and notifications, and (3) contributions to infection control measures using a database of app users’ movement data [15]. The results of this previous study suggested that, in the event of a state of emergency that threatens the nation, the Japanese public prioritizes contributions to public health at the group level (eg, infection control) over risks, such as leaking of an individual’s private data. In light of this hypothesis based on our previous research, we conducted this study to identify how people would rank various app features in terms of how much those features make them want to use the app.

### Interpretation of the Results

In scenario A, smartphone cashback was of low importance compared to the other factors for both the government and business clusters. This is consistent with the results of our previous study. Thus, personal health benefits, such as test appointments and other special rights or privileges, tended to be more important in encouraging active app use and participation than financial benefits, such as a smartphone bill discount for using the app. However, the factor that increased motivation for app use the most was the hope of cutting the number of infections and hospitalizations due to infection in the community by half.

However, the results suggested that concern about the use of personal data collected by the app for some secondary purpose was a negative incentive, which was even more significant toward app use compared to the other 3 factors. People were most likely to feel that they did not want to use the app if there was a chance that their data could be used for secondary purposes unrelated to infection control. The difference in results between the government and private clusters for scenario A demonstrated that people tend to emphasize that the product is for the public good and that personal data should not be used for unscrupulous purposes when they are being managed by a private business.

In scenario B, the most important factor was concern over the risk that the government authority or business would share personal data with a third party. This concern was found to act as a negative incentive much more significantly than the other 3 factors. The type of personal data that the app distributor could use was found to have even less importance than a smartphone bill discount. Citizens seemed to feel minimal concern even when the scope of accessible personal data was expanded to include location and movement. Further, when app users’ personal data were managed by the government, there was a minimal expectation that the data would be used for purposes other than public safety (eg, coupons). In contrast, when the app administrator was a business, there was a minimal expectation that personal data would be used for public safety measures other than infection control.

The importance of profit sharing may have been greater in scenario B than A because the “scope of personal data” and “scope of use” factors from scenario B were found to be the least important among the 7 factors. Although “financial benefit” was set as a common attribute across both scenarios, it cannot be used for statistical adjustment to enable direct comparison of the other 6 factors. However, the results demonstrated relatively extreme differences in importance levels. Thus, if the importance of financial benefits is understood to be perceived in the same way across both scenarios, we believe that it is acceptable to interpret the importance and partworth utilities for all 7 items together. If we do so, the results of this study can be interpreted to suggest that potential app users are most concerned about the risk of personal data being used for purposes other than those specified or being shared with third parties not involved in management, whereas contributing to the public good through app use may be a relatively large incentive.

### Generalizability

The results of this study strongly suggest the need to explore the following points when developing a contact notification or tracing app in anticipation of the next infectious disease pandemic. First, this study demonstrated that to develop a large user base for the app, the entity responsible for managing personal data collected by the app (government and business) must clearly explain that users’ personal data will not be used for unnecessary or unspecified purposes by the managing body, nor will they be shared with a third party without the app user’s consent. The managing body must also make potential app users understand that such risks have been minimized. Second, this study’s results demonstrate that app developers should emphasize features contributing to the public over personal health benefits or financial profit sharing for app users when choosing app user benefits during app development. This factor is also likely to be significant when appealing to the public at large [17]. Third, rather than limiting the scope of the personal information collected, it would be worthwhile to carefully examine the scope of data necessary for preventing the spread of infectious disease and to include these data within the scope of collection after thoroughly explaining this to the public and obtaining their consent. These data could likely include aspects such as app users’ movement data, which were not included in the COCOA app [18].

### Strengths and Limitations

This study offers a conceptual supplement to the results of our 2021 study by incorporating conjoint analysis. While the results can be interpreted in largely the same way, we believe that the results of this study offer clearer suggestions for designing key functions during app development as well as aspects to highlight in conversation with the public when launching a contact tracing app in the future.

However, this study does have some methodological limitations. First, conjoint analysis as a methodology provides limited scientific evidence. It is not possible for attributes to encompass all of an app’s features, and the attributes selected represent no more than a handful of the features that an app possesses. Further, although the scenarios used were relatively generic, they are not completely generalizable. Second, it was necessary to generate numerous attributes for conjoint analysis to achieve our research objectives. We decided that it would not be possible to include all 7 of the attributes (each with 3 levels) that we were focusing on in 1 scenario. We addressed this problem by establishing 2 scenarios, each with 9 experiment protocols. Nonetheless, this design makes it difficult to directly compare the 7 attributes when interpreting the results.

### Conclusions

The results of our conjoint analysis study provide instructive evidence for understanding the relationships between the risk of invasion of personal privacy, health benefits, and financial incentives, as well as their weight in comparison to motivation to contribute to the public good. This information is key when thinking about ethics, law, and social issues in the information age. Moreover, the study offers concrete suggestions for key app functions that anticipate the development of new contact detection and tracing apps. In the future, those responsible must be prudent in handling the risk of invasion of privacy, use of personal data for nonspecified purposes, and third-party data sharing when developing and using relevant apps. Furthermore, the results of this study suggest that potential app users may be positively motivated not only by personal health benefits but also by contributing to public health.

Although these survey results describe the typical thinking of people living in Japan, residents’ attitudes toward ethical, legal, and social issues are strongly influenced by the cultural environment in which they live. Future comparisons with surveys conducted in other Asian and non-Asian countries will further deepen the discussion. We also hope that the methodology used in this study will be used in future studies to describe the balance of values held by people in different ways.

## Appendix

Multimedia Appendix 1 The scenarios used in this survey (government cluster scenarios).

## Abbreviations

CMI: Cross Marketing Inc
COCOA: COVID-19 Contact-Confirming Application
MHLW: Ministry of Health, Labour and Welfare

## Data Availability

Data sharing is not applicable to this paper as no datasets were generated or analyzed during this study
