# Supplementary material for: Survey of Citizens’ Preferences for Combined Contact Tracing App Features During a Pandemic: Conjoint Analysis
Source: JMIR Public Health Surveill. 2024 Nov 14;10:e53340. doi: 10.2196/53340 (PMC11605258; doi:10.2196/53340)
Supplement: Multimedia Appendix 1 [file publichealth_v10i1e53340_app1.docx]

**Appendix: The scenarios used in this survey (government cluster scenarios)**

Please read the following descriptions and answer for each of Cases A and B. Note that each case is hypothetical and does not reflect what is actually being considered. Then, rearrange the attributes according to your preference.

**Case A**

The Ministry of Health, Labour and Welfare is comprehensively reviewing the contact-confirming app that has been used until now to manage the spread of infectious diseases. A new, free app is being developed and distributed, with widespread public use encouraged. In addition to notifying users of contact with confirmed cases, the following are being considered for this app:

Consideration 1: Encouraging widespread app use among the public

Consideration 2: Providing extensive information about the new coronavirus infection via the app

Consideration 3: Obtaining users’ personal information for public health measures

The app will include the following specific attributes to address these considerations.

Attribute A-1 [Profit sharing]: Profit sharing for app users:

a) No financial profit sharing (none)

b) A 500-yen discount coupon per month(500 yen)

c) A 1500-yen discount coupon per month (1500 yen)

Attribute A-2 [Public benefits]: Public benefits of app registration

a) Unknown whether it will benefit infection control measures (unknown)

b) Expected to cut infections by half (infections halved)

c) Expected to cut infections and hospitalizations by half (infections + hospitalizations halved)

Attribute A-3 [Personal health benefits]: Notification of health information and other information to users

a) Immediate notification to the app user if there was close contact with an infected person (contact notification)

b) In addition to a), a priority appointment for a free PCR test (test appointment)

c) In addition to a), a priority appointment for a free test and examination if a user tests positive (test + examination appointment)

Attribute A-4 [Distributor rights]: Authority of the Ministry of Health, Labour and Welfare to use personal information outside of the app:

a) Not authorized to handle personal app user data (No authority)

b) Authorized to use for data analysis in the context of infection control measures only and for notifications to the app user (Infection control only)

c) Authorized to use app user data for broad purposes outside of infection control measures, including advertising (Broad use)

**Case B**

Please treat this case as independent from Case A.

The Ministry of Health, Labour and Welfare is comprehensively reviewing the contact-confirming app that has been used until now to manage the spread of infectious diseases. A new, free app is being developed and distributed, with widespread public adoption being encouraged. In addition to the function of notifying users upon contact with positive cases, the following attributes are being considered. When registering with the app, users must provide their name, mobile phone number, and email address.

Considerations for the app:

Consideration 1: Encouraging citizens to use the app

Consideration 2: Notifying users of the following three pieces of information via the app

(1) The risk of infection in specific locations or areas (ranging from extremely low to fairly high).

(2) If close contact is confirmed, a notification will be sent to encourage testing and specific actions,

(3) If close contact is confirmed, information about priority appointments for testing and medical examinations will be provided.

Consideration 3: Obtaining users’ personal data for public health measures

Consideration 4: Making secondary use of the collected data

The specific attributes of the app to be developed for this purpose are as follows.

Attribute B-1 [Profit sharing]: Profit sharing for app users:

a) No financial profit sharing (none)

b) A 500-yen discount coupon per month (500 yen)

c) A 1500-yen discount coupon per month (1500 yen)

Attribute B-2 [Scope of personal data]: Scope of personal data collected by the Ministry of Health, Labour and Welfare

a) Data from registration only (registration data)

b) Registration data + data concerning location and time of contact with an infected person (contact location data)

c) Registration data + a record of all movements (all movement data)

Attribute B-3 [Scope of use]: Scope of use of collected personal data

a) Planning and implementing measures to prevent the spread of infection (infection control only)

b) In addition to a), for addressing and disseminating information during a state of emergency due to earthquakes or other disasters (infection control

+ other public safety measures)

c) In addition to b), notifications from businesses to app users about useful information and discounts (public safety measures + advertising)

Attribute B-4 [Third party data sharing]: The Ministry of Health, Labour and Welfare provides information on app users to third parties, such as commercial businesses

a) Cannot provide app user data to third parties (data sharing prohibited)

b) Can provide data if anonymized so that individuals cannot be identified (sharing of anonymous data)

c) can provide data that identify individuals (sharing of personal data)
